# Do Parental Pain Knowledge, Catastrophizing, and Hypervigilance Improve Following Pain Neuroscience Education in Healthy Children?

**DOI:** 10.3390/children8050420

**Published:** 2021-05-20

**Authors:** Pere Bacardit Pintó, Kelly Ickmans, Emma Rheel, Margot Iwens, Mira Meeus, Jo Nijs, Roselien Pas

**Affiliations:** 1 Pain in Motion Research Group (PAIN), Department of Physiotherapy, Human Physiology and Anatomy (KIMA), Faculty of Physical Education & Physiotherapy, Vrije Universiteit Brussel (VUB), Laarbeeklaan 103, 1090 Brussels, Belgium; pere.bacardit.pinto@vub.be (P.B.P.); Emma.Rheel@vub.be (E.R.); Margot.Paula.Iwens@vub.be (M.I.); jo.nijs@vub.be (J.N.); Roselien.pas@vub.be (R.P.); 2 Department of Physical Medicine and Physiotherapy, University Hospital Brussels, 1090 Brussel, Belgium; 3 Research Foundation—Flanders (FWO), Egmontstraat 5, 1000 Brussel, Belgium; 4 Department of Experimental-Clinical and Health Psychology, Ghent University, Henri Dunantlaan 2, 9000 Gent, Belgium; 5 Pain in Motion International Research Group; Mira.meeus@uantwerpen.be; 6 MOVANT Research Group, Department of Rehabilitation Sciences and Physiotherapy, Faculty of Medicine and Health Sciences, University of Antwerp (UA), 2610 Wilrijk, Belgium; 7 Department of Rehabilitation Sciences and Physiotherapy, Faculty of Medicine and Health Sciences, Campus UZ, Ghent University, 9000 Ghent, Belgium; 8 Unit of Physiotherapy, Department of Health and Rehabilitation, Institute of Neuroscience and Physiology, Sahlgrenska Academy, University of Gothenburg, 405 30 Gothenburg, Sweden

**Keywords:** pain neuroscience education, healthy children, parents, pain knowledge, fear of pain, pain catastrophizing

## Abstract

Pediatric chronic pain is a challenging problem for children and their families, although it is still under-recognized and under-treated. The aim of this study was to investigate whether a pain neuroscience education program for children (PNE4Kids) delivered to healthy children aged 8 to 12 years old and attended by their parents would result in improved parental knowledge about pain neurophysiology, decreased parental pain catastrophizing about their own pain and their children’s, decreased parental pain vigilance and awareness, and decreased fear of pain in children. Twenty-seven healthy child–parent dyads received a 45 min PNE4Kids session. Demographic data were collected, and the Neurophysiology of Pain Questionnaire (NPQ), Fear of Pain Questionnaire—Parent Proxy Report (FOPQ-P), Pain Catastrophizing Scale (PCS), Pain Catastrophizing Scale for Parents (PCS-P), and the Pain Vigilance and Awareness Questionnaire (PVAQ) were completed by the parents before and after the PNE4Kids session. Twenty-six dyads completed study participation. In response to the PNE4Kids session, significant short-term (1 week) improvements were shown in the NPQ (*p* < 0.001) and the FOPQ-P (*p* = 0.002). Parents’ level of pain knowledge and children’s fear of pain, reported by their parents, improved after a 45 min PNE4Kids session. Thus, PNE4Kids should likewise be further investigated in healthy child–parent dyads as it might be useful to target parental and children’s pain cognitions at a young age.

## 1. Introduction

Research regarding pediatric chronic pain is increasing as it appears to be a challenging clinical problem for children and their families. However, the topic is still under-recognized and under-treated [1,2,3]. Around 30% of the pediatric population suffers from chronic pain, with headache, abdominal pain, and musculoskeletal pain being the most prevalent types [4,5,6,7,8,9,10]. Moreover, pediatric chronic pain has been found to be more prevalent in girls [4]. Children with chronic pain are more likely to experience social, academic, and recreational difficulties as well as impaired family functioning [2,4,6,11]. In addition, individuals experiencing chronic pain during childhood have a higher risk of suffering from chronic pain during adulthood [5,12].

Looking at these epidemiologic data, it is clear that prompt action should be taken. As proposed by Louw et al. [13], one action to decrease the prevalence of pediatric chronic pain and its consequences is prevention. Prevention can be performed through pain neuroscience education (PNE) as it teaches people about the true function of pain and eventually aims to decrease the threat value of pain, which will hypothetically result in adaptive beliefs and cognitions, adequate coping strategies, and behavioral responses [13]. 

Thus, PNE might contribute to more adequate coping with future pain situations and might minimize the transition from acute pain to chronic pain, given that emotions, cognitions, and behaviors related to pain are among the wide variety of risk factors for this transition [13,14]. Indeed, a child’s pain experience has been found to be related to the child’s fear of pain and previously developed pain memories, as well as to parental anxiety or magnification thoughts about pain [2,15]. Research has also shown that parental psychological flexibility and certain behaviors affect the child’s pain perception, coping, and functioning [15,16,17,18,19]. 

PNE4Kids is a PNE program that has recently been developed for children aged 6–12 years old and uses an interactive board game to explain pain science to children [5,6]. PNE4Kids has been tested in children suffering from functional abdominal pain disorders (FAPD), showing that a combination of a single session of PNE4Kids and hypnotherapy was able to reduce parental pain catastrophizing and improve children’s functional disability [20]. During the PNE4kids session, the influence of pain beliefs, cognitions, and behaviors on the child’s pain is discussed. Although the intervention was directed to the child with FAPD, parents also attended the PNE4Kids intervention and were given a chance to ask content-related questions at the end of the session. This intent of parent involvement might have led to improved parental outcomes, such as parental pain-related attitudes, protective behavior in response to their child’s pain, and perceptions of the child’s functioning [6,7,15,17]. A better understanding of their child’s pain may lead to adequate parental supportive behavior based on the rationale that the child’s pain behaviors can be driven by their parents or the caregivers [20]. Thus, it is crucial to include parents or caregivers as part of the prevention process and track potential changes in their pain behavior.

To date, only one study investigated changes associated with PNE, which was delivered using a 30 min PowerPoint presentation to middle school students aged 10–15 years in a school environment. The study demonstrated a positive shift in the student’s pain knowledge as well as healthier beliefs regarding pain. It was the first study to show that PNE can be delivered as a preventive strategy being performed in a mixed sample of healthy individuals and individuals with pain [13]. 

The aim of this study was to examine if a single session of PNE4Kids [5] delivered to healthy children aged 8 to 12 years old and attended by their parents is accompanied by improvements in parental knowledge about pain neurophysiology, decreased parental pain catastrophizing about their own pain and their children’s pain, decreased parental pain vigilance and awareness, and decreased fear of pain in children reported by their parents.

## 2. Materials and Methods

### 2.1. Study Design and Setting 

This study is an exploratory study examining the short-term changes observed after giving a single individual PNE4Kids session to a sample of healthy child–parent dyads and took place in Flanders, Belgium. The study protocol was first registered at clinicaltrials.gov on May 2017 (NCT03164343) and received approval from the Ethics Committee of the Antwerp University Hospital (B300201732479). Appendix A presents information regarding the original focus of the study as compared with the data presented herein. Prior to participant enrollment, written informed consent was obtained from the co-participating parent/guardian, as well as from the child (in case the child was 12 years old). 

### 2.2. Participants and Study Flow

Eligible participants were healthy children between 8 and 12 years old and their parents. Exclusion criteria were the following: children who (1) previously received any form of an education session specifically focused on pain; (2) have or have had chronic pain defined as pain persisting for three months or longer; (3) are not native Dutch speakers; (4) have a cognitive-developmental impairment, as it could compromise the understanding of the neurophysiology of pain. No exclusion criteria were added regarding the parents given the focus of the intervention on children and also because the initial main goal was to study the children’s pain knowledge via the Pediatric Neurophysiology of Pain Questionnaire (PedNPQ; see Appendix A). Parent–child dyads were recruited via announcements in several primary schools in Flanders and via social media. Potential participants were assessed via a telephone interview for possible exclusion criteria. All eligible participants were enrolled in the study. After study eligibility was assessed and informed consent was obtained, children received one PNE4Kids session at their home, which was attended by at least one parent (the one filling out the questionnaires).

Measurements were conducted at baseline (T1) and after the intervention (T2). T1 assessments included (1) patient demographics, (2) questionnaires about parents’ neurophysiology of pain knowledge (Neurophysiology of Pain Questionnaire (NPQ)), catastrophic thinking about pain (regarding their own pain (Pain Catastrophizing Scale (PCS)) as well as their child’s pain (Pain Catastrophizing Scale for Parents (PCS-P)) and pain vigilance and awareness (Pain Vigilance and Awareness Questionnaire (PVAQ)), and (3) a parent proxy report about the child’s pain-related fear (The Fear of Pain Questionnaire—Parent Proxy Report (FOPQ-P)). T2 assessments took place one week after the PNE4Kids session and, besides the demographic questionnaire, included the same questionnaires as at T1.

### 2.3. Intervention

Children received a 45 min PNE4Kids session, which was delivered individually to the child and attended by their parent(s). Throughout the educational session, an interactive board game was used to ensure interaction between therapist and child. Parent(s) were present during the PNE4Kids session and were offered the possibility to ask questions at the end of the program. The PNE4Kids session contained themes such as central nervous system anatomy, nociception, peripheral and central sensitization, bio-psycho-social factors associated with pain, threat appraisal of the brain, stress, and endocrine responses in pain, as well as various strategies to ease pain. For more information regarding the development and feasibility of the PNE4Kids program, we refer the interested reader to Pas et al. [5]. The content of PNE4Kids can be freely accessed via http://www.paininmotion.be/pne4kids. All sessions were provided by the same physical therapist (R.P.), trained in providing PNE4Kids to children.

### 2.4. Measures

#### 2.4.1. Demographics

Basic demographic (e.g., child’s and parent’s age and gender, and child’s ethnicity) and additional information (e.g., parent’s educational level, parent’s previous experiences with PNE, and parent’s own experience of chronic pain) was obtained through the children’s and parent’s report. 

#### 2.4.2. Primary Outcome 

The Dutch version of the Neurophysiology of Pain Questionnaire (NPQ) was used to assess parental pain knowledge, and consists of 19 items. Each item is rated as “true,” “false,” or “undecided.” Each correct answer was counted as one point, resulting in a maximum score of 19. This questionnaire is able to detect the effects of cognitive interventions in a clinical and research setting [21]. The NPQ is validated both in English and Dutch [22,23,24,25]. The NPQ has a person separation index (PSI) of 0.84 (Cronbach’s alpha equivalence given by the Rasch analysis for internal consistency), and a good test–retest reliability with an ICC score of 0.971 (95% confidence interval [CI]: 0.925–0.987) (before education) and an ICC score of 0.989 (95% CI: 0.981–0.984) (after education) [22]. 

#### 2.4.3. Secondary Outcome Measures

The Fear of Pain Questionnaire—Parent Proxy Report (FOPQ-P) was used to assess children’s fear and avoidance reported by their parents. The Dutch version of the FOPQ-P was adapted from the parent report of the validated Fear of Pain Questionnaire. The FOPQ-P has 23 items with a Cronbach’s alpha of 0.92 for the overall scale [26].

Parental catastrophic thinking about their own pain was measured using the Dutch version of the Pain Catastrophizing Scale (PCS). The Dutch translation of the Pain Catastrophizing Scale (PCS) is a widely used measure for the assessment of pain catastrophizing and has established clinometric properties. It consists of 13 items describing different thoughts and feelings that could be experienced in relation to their own pain. Parents rate how frequently they experience each of the thoughts and feelings when they are in pain using a 5-point scale (0 = ‘not at all’, 4 = ‘extremely’). The scale yields a total score between 0 and 52 [27]. The scale has good internal consistency (Cronbach’s alpha of 0.92, 95% CI 0.91–0.93) and test–retest reliability (Spearman rho of 0.88, 95% CI 0.83–0.93) [28]. 

Parental catastrophic thinking about their child’s pain was measured using the Dutch version of the Pain Catastrophizing Scale for Parents (PCS-P) [29]. The PCS-P questionnaire contains 13 items, describing different catastrophic thoughts and feelings that parents may experience in relation to their child’s pain [29]. Parents rate how frequently they experience each of the thoughts and feelings when their child is in pain using a 5-point scale (0 = ‘not at all’, 4 = ‘extremely’). Just as the PCS, the scale yields a total score between 0 and 52. The developer reported strong internal consistency (overall scale Cronbach’s alpha of 0.93) and validity as demonstrated by associations with parent distress and child disability [29]. 

Parental attention to pain was measured using the Dutch version of the Pain Vigilance and Awareness Questionnaire (PVAQ). The PVAQ contains 9 items and presents a good internal consistency reliability (overall scale Cronbach’s alpha of 0.83, reported by previous research). It aims to measure the attention that individuals present towards their pain [30,31].

The internal consistency for this study sample can be found in the results section.

### 2.5. Sample Size 

The sample size calculation was performed using G*Power and based on the effect sizes on our primary outcome measure pain knowledge measured with the NPQ. Since no study has ever reported on the pain knowledge of parents after a single PNE intervention delivered to their child, we looked at the effect size of one study assessing pain knowledge in children after a single PNE session delivered to the child [13], and two other studies assessing pain knowledge in adults with chronic fatigue syndrome (CFS) [32] and fibromyalgia [33] after a single PNE session delivered to the adult. These studies provided us with effect sizes between d = 1.7 and d = 2.8 for pain knowledge measured with the NPQ immediately measured after 1 session of PNE. Using the most conservative effect size of d = 1.7, a power of 0.95, two-tailed testing, and alpha = 0.05, the calculation resulted in a total sample size of 7 participants. Accounting for possible missing data and keeping in mind that every sample size calculation has its limitations, we aimed to recruit 27 participants to be able to obtain analyzable data of at least 20 participants.

### 2.6. Statistical Analyses 

The statistical analyses were performed using IBM SPSS Statistics for Macintosh, Version 25.0. (IBM Corp. Released 2017. Armonk, NY, USA). The significance level was set at α < 0.05. Descriptive statistics of numbers and percentages were used to report the demographics of the sample. The Shapiro–Wilk test and the assessment of Q–Q plots and histograms showed that all the data followed a normal distribution except for the PCS data (Shapiro–Wilk *p* = 0.010). Internal consistency of the data obtained using the different questionnaires was examined by calculating Cronbach’s alpha coefficient for both assessments (i.e., baseline and post-education).

In order to evaluate the changes associated with the PNE4Kids session on parental pain-related outcomes, a paired sample *t*-test was performed to compare baseline and after-treatment scores for each variable. For the PCS scores, the Wilcoxon signed-rank test was used. The effect size was calculated by the formula of Rosenthal because data followed normal and non-normal distributions (PCS), which violated the general assumptions of Cohen’s formula [34,35]. 

## 3. Results

### 3.1. Demographic Data

A total of 27 child–parent dyads agreed to be enrolled in the study. During the study, there was one incomplete case due to a parent who forgot to fill in the questionnaires. Finally, the results of 26 dyads (81% mothers, average child age 9.81 ± 1.33 years) were analyzed. All of the participants were Caucasian, with 96% of the participants coming from the Flemish Brabant region and the other 4% coming from the West Flanders region. Demographics both for the parents and children are reported in Table 1. 

### 3.2. Internal Consistency

The Cronbach’s alpha for each questionnaire can be found in Table 2.

### 3.3. Outcomes Pre- and Post-Intervention

Following the intervention, NPQ scores increased significantly (*p* < 0.001) and FOPQ-P scores decreased significantly (*p* = 0.002). For the PCS-P, PCS, and PVAQ, no significant differences were found following the intervention (*p* > 0.05). Mean scores of the baseline and post-intervention outcomes are reported in Table 3.

Changes in NPQ: 70.37% of the participants improved their score; 18.52% maintained the same score and 11.11% showed a decrease in pain knowledge score. At T2, the mean pain knowledge score improved by 15.38%. The NPQ questions for which the biggest change (≥10 participants changed their answer) was observed after the intervention are shown in Table 4; showing the number of people who changed their responses and the percentage of people who changed to the correct answer.

### 3.4. Post-Hoc Analysis

Parent education level was further investigated to check if there was any relation with the scores obtained in the different variables. After performing ANOVA analysis and the Kruskal–Wallis test for the non-parametric data (PCS), FOPQ-P scores appeared to be influenced significantly by parent education level. The post hoc test showed that parents with a bachelor’s degree reported significantly lower child’s fear and avoidance behavior in comparison with parents with a high school degree (*p* = 0.031 for the 1st assessment and *p* = 0.044 for the 2nd assessment). In addition, at the first assessment, graduates reported significantly lower fear and avoidance behavior in their children in comparison with parents with a high school degree (*p* = 0.038).

In order to improve the interpretation of the results, a post hoc power analysis for each of the secondary outcome measures was performed when comparisons were not significant. The post hoc power analyses revealed low power for all the non-significant pre-post comparisons (i.e., PCS-P (50%), PCS (45%), and PVAQ (22%)).

## 4. Discussion

This study aimed to explore changes in parental pain knowledge and pain-related cognitions and children’s pain-related cognitions reported by the parents associated with a single PNE4Kids session of 45 min that was delivered individually to healthy children and attended by their parents.

In the current study, parents’ level of pain knowledge and children’s fear of pain reported by their parents improved significantly in the short term after a 45 min PNE session. Indeed, after PNE4Kids, parents reported that their children were showing less fear and avoidance behavior related to pain; possibly meaning that their child is avoiding fewer activities or appears to be less fearful of painful events during the week after the PNE4Kids session. Changes in parents’ pain appraisals might be beneficial for children as they are shown to modify their child’s pain perception, coping, and functioning [9,15].

The internal consistency of the used measures in our study sample was good to excellent, with Cronbach’s alpha coefficients ranging from 0.766 to 0.952. This is in line with previous work supporting the internal consistency of these measures in other populations [22,26,28,29,31], and supports the validity of the study findings.

The positive results of our study are in line with previous studies looking at the changes associated with PNE for children. Louw et al. (2018) [13] delivered PNE via a PowerPoint presentation to a group of middle school students (mean age of 12.74 (SD 1.13) years), including healthy children and children experiencing pain; showing a general improvement in pain knowledge and pain beliefs after attending a 30 min PNE lecture.

One recent study investigated the effectiveness of PNE4Kids in combination with hypnotherapy in children with FAPD [20]. The scores on the Pain Catastrophizing Scale-Parent (PCS-P), the Faces Pain Scale-Revised (FPS-R), pressure algometry, and conditioned pain modulation obtained from the group who received the combination of the two therapies did not reach statistical signification when compared to the scores obtained by the group who received hypnotherapy alone. However, the combination improved pain catastrophizing and functional disability. As this is the only study that evaluates PNE4Kids in a sample of the same age as in our study, both studies show that PNE4Kids is safe and is associated with positive changes in pain-related behavior in children from 8–12 years old as reported by their parents.

The biggest changes in parental NPQ scores observed in our study were linked to questions referring to nerve function, descending pain modulation, pain processing, and pain origin. The fact that these questions showed more variations could indicate that these topics are either the ones less known or the ones linked with stronger societal misconceptions about pain. The high percentage of people changing their answers to choose the correct option shows the ability of the PNE4Kids to cover these topics successfully. Some of these questions are stated to be more difficult and challenging than others present in this questionnaire [22].

Although parental knowledge about pain neurophysiology (NPQ) and fear of pain in children reported by their parents (FOPQ-P) scores improved, variation in parental pain catastrophizing about their own pain and their children’s pain (PCS, PCS-P) and parental pain vigilance and awareness (PVAQ) scores was not significant. Several reasons, which can be seen as limitations of this study, might explain this lack of change over time. First, children were healthy, and the initial scores presented by their parents for these variables were low compared with parents of children experiencing chronic pain (PCS-P: 15.67 (SD 9.90); PCS: 20.3 (SD 11.5); and PVAQ: 40.0 (SD 12.1)) [29,31]. Second, the lack of extra sessions for feedback implies fewer opportunities to generate substantial changes in terms of education. Performing a follow-up may also help tailor the remaining incorrect beliefs and cognitions regarding pain through repetition and further discussion. Third, in this study, we evaluated only short-term changes, while significant changes in these outcomes might have appeared later in time as pain knowledge becomes more integrated into their everyday lives. Finally, low power for all the non-significant pre-post comparisons was shown by the post-hoc analyses. This means that ‘false negative’ results, probably as a result of the small sample size, cannot be excluded in these cases.

Our results are in line with the findings obtained by Reid and colleagues [3], who developed an art narrative-based e-book for parents with children with chronic pain. In their study, parents’ pain knowledge increased up to 21.4% and their confidence in responding to some of the knowledge questions increased as well, showing that PNE delivered with the help of a tool is helpful for parents. The group of parents also agreed that they would recommend it to other families in a similar situation. The study of Reid and colleagues [3] and our study show how useful it can be to use and adapt tools to deliver PNE to parents.

To our knowledge, this is the first study examining whether a single session of PNE4Kids delivered to healthy children aged 8 to 12 years and attended by their parents is accompanied by improvements in parental pain knowledge. Another important strength of this study is that, with 26 child–parent dyads, the study was sufficiently able to detect a statistically significant within-group difference in parental pain knowledge.

Moreover, the study also presents some limitations that should be taken into account when interpreting its results. In total, 88.5% of the parent sample in this study has a higher education diploma. This level of education does not accurately reflect the actual social situation, and in future research, it would be interesting to see whether findings would be similar if the study was carried out in children with parents with a lower education level. As shown by the post hoc analysis, higher parental education level was related to lower scores in FOPQ-P; therefore, in future studies, parental education level should be further investigated. Second, it could have been interesting to include more information about the child; for example, regarding the child’s catastrophic thinking about pain with the Pain Catastrophizing Scale for Children (PCS-C) [36], and children’s pain knowledge with the Concept of Pain Inventory (COPI) [37]. The Concept of Pain Inventory (COPI) was published after the study was performed, and it is a good option for assessing a child’s pain concept in future studies. Third, selection criteria were formulated for the children; future studies could include selection criteria for the parents as well. Finally, the inclusion of a longer follow-up period to investigate whether changes that appeared in the short-term would be retained after mid- and long-term follow-up and whether other changes would occur later would be interesting for future research.

In conclusion, a single 45 min session of PNE4Kids was associated with a significant increase in parental pain knowledge as well as a significant decrease in parental proxy reports about their child’s fear of pain in the short term. Based on these results, this type of intervention could be a strategy to prevent the transition from acute pain to chronic pain in children, given that a child’s pain experience is related to parental anxiety or magnification thoughts about pain [13,14]. To the best of our knowledge, this is the first study examining the parental response to a PNE program targeting young, healthy children (8–12 years). Future controlled studies with bigger samples should investigate (1) the evolution of changes in the long term, (2) the applicability of PNE4Kids to other beneficiaries (adults and children), and (3) preventive effects of delivering PNE to children on the development of chronic pain in adulthood. In order to do this, randomized controlled trials with appropriate control interventions are needed to examine the possible effects of PNE4Kids in healthy children and their parents. If future studies show mid-term and long-term benefits, PNE4Kids should be implemented as early as possible with several sessions and at a large scale, for example, in the school curriculum.

## Figures and Tables

**Table 1:** Demographic data (*n* = 26).

**Parents**	
Sex of parent (*n* (%))	
• Male	5(19.2)
• Female	21(80.8)
Age of parent, years (mean (SD))	49.19 (5.18)
Educational level of parent (%)	
• Doctorate	3.8
• Master’s	46.2
• Bachelor’s	38.5
• High school	11.5
Previous or ongoing chronic pain (*n* (%))	
• Yes	4 (15.4)
• No	22 (84.6)
**Children**	
Sex of child (*n* (%))	
• Male	12 (46.2)
• Female	14 (53.8)
• Age of child, years (mean (SD))	9.81 (1.33)

**Table 2:** Pre-and post-intervention Cronbach’s alpha coefficients.

Test	Pre-Intervention Cronbach’s Alpha	Post-Intervention Cronbach’s Alpha
Primary outcome		
NPQ	0.773	0.813
Secondary outcomes		
FOPQP	0.925	0.952
PCS-P	0.856	0.827
PCS	0.858	0.889
PVAQ	0.776	0.842

**Table 3:** Outcomes pre-and post-intervention.

Test	Pre-Intervention (Mean (SD))	Post-Intervention (Mean (SD))	r	*t*-Value	*p*-Value
Primary outcome					
NPQ	9.46 (3.679)	12.38 (3.047)	0.637	−4.554	0.000 **
Secondary outcomes					
FOPQP	34.38 (14.749)	28.65 (16.270)	0.563	3.403	0.002 *
PCS-P	11.81 (6.735)	10.23 (5.309)	0.267	1.385	0.178
PCS ^a^	9.50 (10.250)	6.50 (6.750)	0.352	1.965	0.073
PVAQ	31.35 (8.908)	29.62 (9.884)	0.329	1.740	0.094

*: *p*-value <0.01. **: *p*-value <0.001. r: Effect size. ^a^: As the data were following a non-normal distribution, the median (IQR) was reported. NPQ: Neurophysiology of Pain Questionnaire, FOPQP: Fear of Pain Questionnaire—Parent Proxy Report, PCS-P: Pain Catastrophizing Scale for Parents, PCS: Pain Catastrophizing Scale, PVAQ: Pain Vigilance and Awareness Questionnaire.

**Table 4:** Variations in NPQ answers after intervention with PNE4Kids.

N°	Question	N° Changes	% Changed to Correct
4	“Special nerves in your spinal cord convey ‘danger’ messages to your brain.”	15	60.00
6	“Pain occurs whenever you are injured.”	14	92.86
7	The brain sends messages down your spinal cord that can change the message going up your spinal cord.”	10	90.00
8	“The brain decides when you will experience pain.”	11	90.91
10	Chronic pain means that an injury hasn’t healed properly.	13	61.54
12	“Nerves can adapt by producing more receptors.”	14	78.57
14	“Nerves adapt by making ion channels stay open longer.”	11	63.64

NPQ: Neurophysiology of Pain Questionnaire. N°: Number of the question. N° Changes: Number of people who changed their answer after receiving PNE4Kids. % Changed to correct Percentage of N° Changes (people who changed their answer after receiving PNE4Kids) to a correct answer.

## Data Availability

The data presented in this study are available on request from the corresponding author. The data are not publicly available due to privacy reasons.

## Appendix A

For transparency, the following explains the differences between our study protocol and the current work. The original protocol focused on studying the variation in the PedNPQ generated by the PNE4Kids session. The PedNPQ was developed to assess pain knowledge in young children. When testing the test–retest reliability of the newly developed PedNPQ questionnaire, the 95% confidence interval of the ICC estimate for the total score of the PedNPQ was 0.32–0.62, indicating a level of reliability as “poor” to “moderate” and Cohen’s Kappa of each PedNPQ item ranged from 0.200 to 0.489 showing a “slight” to “moderate” reliability. As the questionnaire appeared to not be valid, we focused our study and analysis on the rest of the variables.

In future studies assessing PNE in pediatrics, the Concept of Pain Inventory (COPI) could be a good option to assess child’s concept of pain. It shows acceptable internal consistency (α = 0.78) and moderate test–retest reliability (intraclass correlation coefficient (3,1) = 0.55; 95% CI, 0.37–0.68) [37].
